# Chemerin rs17173608 and vaspin rs2236242 gene variants on the risk of end stage renal disease (ESRD) and correlation with plasma malondialdehyde (MDA) level

**DOI:** 10.1080/0886022X.2018.1459698

**Published:** 2018-04-12

**Authors:** Hamid Nomani, Hamid Khanmohamadian, Asad Vaisi-Raygani, Ebrahim Shakiba, Maryam Tanhapour, Zohreh Rahimi

**Affiliations:** a Department of Clinical Biochemistry, Kermanshah University of Medical Sciences, Kermanshah, Iran;; b Fertility and Infertility Research Center, Kermanshah University of Medical Sciences, Kermanshah, Iran;; c Medical Biology Research Center, Kermanshah University of Medical Sciences, Kermanshah, Iran

**Keywords:** End-stage renal disease, chemerin, vaspin, single nucleotide polymorphism, tetra primer-ARMS-PCR, malondialdehyde (MDA), oxidative stress

## Abstract

**Introduction:** End-stage renal disease (ESRD) is associated with critical kidney illness that seriously affects the lifespan. Genetic factors and oxidative stress could play critical role in the development of ESRD. We assessed the association between chemerin rs17173608 T/G and vaspin rs2236242 T/A genes variants with the risk of ESRD and their correlation with plasma malondialdehyde (MDA) level.

**Materials and methods:** In a case-control study, 131 gender and age-matched unrelated healthy controls and 110 ESRD patients were enrolled. The chemerin rs17173608 T/G and vaspin rs2236242 T/A were detected by Tetra primer-amplification refractory mutation system-polymerase chain reaction (T-ARMS-PCR). The MDA concentration was determined by HPLC.

**Results:** Our findings for the first time revealed that in codominant genetic model (T/G vs. T/T genotype), the T/G genotype of chemerin gene significantly had a protective role against ESRD susceptibility. Also, in the presence of chemerin G allele, the risk of ESRD decreased by 0.79-fold (*p* = .048) in Kurdish population of Iran. The MDA serum levels in ESRD patients carrying the chemerin T/G + G/G genotype of rs17173608 T/G and also in carriers of A/A + T/A genotype of vaspin rs2236242 T/A were significantly higher compared to those in control subjects. The overall distribution of vaspin rs2236242 T/A genotypes and alleles comparing ESRD patients and healthy subjects were not statistically significant.

**Conclusion:** We found that the G allele of chemerin rs17173608 compared to T allele decreased the risk of ESRD, and there was a significant association between chemerin and vaspin variants with plasma MDA level in a sample of the Iranian population.

## Introduction

Several conditions [1] such as failure to remove metabolic end-products from the blood, electrolyte and progressive decrease in glomerular filtration rate (GFR) resulted in ESRD, the last stage of chronic kidney disease [2]. The prevalence of ESRD continues to increase in most countries; ∼2000 per million populations (pmp) and 1500 pmp of the adult populations in the Japan and the USA, respectively [3]. The occurrence of ESRD among Iranian population in 2008 was reported to be 466 pmp based on the management center of transplantation and special diseases (MCTSD) criteria [1]. ESRD is likely to be influenced by environmental factors, such as sedentary lifestyle and increased calorie intake, in combination with an unfavorable genotype [4,5]. Furthermore, genetic polymorphisms within the chemerin (tazarotene-induced gene 2 protein (TIG2) or retinoic acid receptor responder protein 2 (RARRES2 or TIG2) have been associated with the basic components of ESRD [6]. Recently, chemerin was found to be highly expressed in liver, adipose tissue, fibroblasts and platelets [7,8]. Chemerin, a novel adipokine and a chemo-attractant protein is involved in adipogenesis, obesity, adipose cell function, maintenance of homeostasis and the activation of natural killer cells, macrophages and dendritic cells in both innate and adaptive immunity [9]. It also stimulates extracellular signal-regulated kinase 2 (ERK1/2) and lipolysis in the 3T3L1 adipocytes, and is an agonist of the orphan G-protein coupled receptor (GPCR), with other unknown biological functions [10]. Chemerin receptor, ChemR23 or CMKLR1 (chemokine-like receptor 1) [11], a Gαi protein linked receptor, is expressed by components of the innate immune system, including tissue-resident macrophages [12,13]. Therefore, some reports suggest an anti-inflammatory function for chemerin [14]. In fact, activated inflammatory mediator cells release the enzymes, which stimulate transformation of circulating prochemerin into chemerin that is essential for recruitment of leukocytes and other cells to sites of inflammation and increase their adhesion [15]. It has been previously reported that in patients with rheumatoid arthritis, ulcerative colitis, Crohn’s disease, psoriasis, chronic pancreatitis and liver diseases, plasma chemerin levels have elevated [16]. In addition, decreased rate of glucose uptake, impaired glucose homeostasis and insulin resistance are linked with chemerin [17]. As the chronic kidney disease progresses, particularly in chronic inflammatory state, glucose and lipid metabolism disorders are observed [18] and high plasma level of chemerin associated with renal dysfunction has been reported by Leiherer [19]. Vaspin (visceral adipose tissue-derived serine protease inhibitor), is a member of the serpin family, which is also known as a serpin peptidase inhibitor, isolated from the visceral adipose tissue (VAT) [20]. Many clinical and experimental studies suggested that serpins had important physiological functions such as blood coagulation, inflammation and host defense, fibrinolysis, and ischemia protection [21]. Previous studies have reported that serum vaspin levels have decreased in the Japanese chronic hemodialysis (HD) patients. An inverse association was found between creatinine and vaspin levels in HD patients [22]. The generation of oxidative compounds play crucial role in inflammation and tissue repair process. ESRD patients are characterized by an imbalance between pro‐oxidant and anti‐oxidant factors [23], and increased oxidant stress has been associated with typical complications of ESRD such as atherosclerosis and β2‐microglobulin amyloidosis that was reported by Locatelli [24]. MDA as a lipid peroxidation product, partially excreted by kidney [25]. MDA is commonly used as a biomarker for lipid oxidative damage recognition [26]. It exists in two different states that are free and bound to proteins, nucleic acids and lipoproteins, which are designated as MDA adducts [27]. Cell membrane lipids are one of the most important target molecules, which can be attacked by the free radicals and MDA that is associated with exacerbating the oxidative damage in various diseases such as cancer, atherosclerosis, Alzheimer’s disease, lupus, preeclampsia, abuse patients, diabetes mellitus, psoriasis and autoimmune diseases [28–32].

Moreover, Plasma MDA level is a strong predictor of cardiovascular disease prevalence in ESRD patients [27]. To the best of our knowledge, there is no report regarding chemerin and vaspin gene variants with ESRD and its correlation with plasma MDA level. The present study aimed to investigate the possible association between chemerin rs17173608 and vaspin rs2236242 gene variants with ESRD and their correlation with plasma MDA level in Kurdish population of Iran.

## Materials and methods

### Individuals and study design

In a case-control study, 131 Iranian healthy control volunteers and 110 ESRD patients undergoing hemodialysis therapy were enrolled. The process of sampling was suggested by the nephrologists and informed consent was obtained from all individuals. Five milliliters blood sample were taken after an overnight fasting, before dialysis, and poured in vials containing EDTA. The samples were transferred to the laboratory and kept at −20 °C. Blood plasma was isolated from the samples for the determination of MDA by HPLC [33,34]. Genomic DNA was extracted from the peripheral blood samples using standard phenol-chloroform and proteinase-K extraction method [35].

### Genotyping and T-ARMS-PCR

The tetra primer-amplification refractory mutation system-polymerase chain reaction (T-ARMS-PCR) method was effectively applied for genotyping rs17173608 T/G and rs2236242 T/A variants of chemerin and vaspin, respectively. This method is simple, rapid, sensitive, reproducible, inexpensive for detection of variants, and does not require special equipment. Both wild-type and rare alleles, were simultaneously amplified together with a control fragment, in a single PCR tube, a distinguishing feature from conventional ARMS-PCR that amplifies bi-alleles in two separate reactions [35,36]. The genotypes determined by this method are in concordance with those determined by sequencing. The chemerin and vaspin genomic sequences (NT_007914.15 and NT_026437.13, respectively) were obtained from the National Center for Biotechnology Information (NCBI) (http://www.ncbi.nlm.nih.gov). Chemerin and vaspin gene variants were used by T-ARMS-PCR method using two external primers and two allele-specific internal primers designed previously and reported by Hashemi et al [37] (Table 1).

**Table 1.** The used primers for detection of single-nucleotide polymorphisms in chemerin rs17173608 and vaspin rs2236242 genes.

Gene variant	Primers	Sequence (5′ to 3′)
Chemerin rs17173608	FI (G allele)	ATTGCTATAGTCCAGTGCCCTTCG
	RI (T allele)	CCAGTTCCCTCTGTCGGCTTAA
	FO	GTCAGACCCATGCAGTTTTCAAAC
	RO	GAGTTCCTCTCTCAAGCATCAGGG
	FI (T allele)	AAGACGCCGCTTCTGTGCACT
Vaspin rs2236242	RI (A allele)	CACAGGGACCCAGGATAACTTGCT
	FO	GGAGGCAGACCAGGCACTAGAAA
	RO	ACCATCTCTCTGGCTTCAGGCTTC

FI: forward inner; RI: reverse inner; FO: forward outer; RO: reverse outer.

PCR method was done using commercially available PCR premix (AccuPower PCR PreMix; BIONEER, Daejeon, Korea) based on the manufacturer’s instructions. Briefly, 1 μL template DNA (∼100 ng/μL), 1 μL of each primer (10 pmol/μL) and 15 μL DNase-free water were added to AccuPower PCR PreMix. Amplification was performed with an initial denaturation at 95 °C for 5 min, followed by 30 cycles of 30 s at 95 °C, 15 s at 65 °C for chemerin, 30 s at 62 °C for vaspin, respectively, and 30 s at 72 °C with a final step at 72 °C for 10 min. PCR products verified on a 2.0% agarose gel contained 0.5 μg/mL ethidium bromide and photographs were taken. To ensure T-ARMS-PCR genotyping quality, we genotyped all variants in random samples and no genotyping mistake was found.

### Malondialdehyde assay

Butylated hydroxytoluene (BHT), MDA, methanol, 2-thiobarbituric acid (TBA) and 1,1,3,3-tetraethoxypropane (TEP) were of analytical grade and purchased from Sigma-Aldrich Co. (St. Louis, MO, USA). All other reagents were products of Merck (Darmstadt, Germany). Plasma MDA was measured by high-performance liquid chromatography (HPLC; Agilent, Boblingen, Germany) using an EC 250 /4.6 Nucleodur 100–5 C18ec column (Macherey-Nagel, Duren, Germany). A plasma or TEP standard (50 μL of a stock standard solution containing 5 μmol L^−1^ TEP in 40% ethanol solution) was mixed with 50 μL BHT (0.05% *v̸v* BHT in ethanol), 400 μL H3PO4 and 100 μL TBA (42 mmol L^−1^ in 0.44 mol L^−1^ H3PO4) and incubated at 100 °C for 1 h. Following heat derivatization, samples were cooled on ice for 10 min. The MDA–TBA complex was then extracted from the mixture with *n*-butanol (250 μL). The tubes were vortex mixed for 5 min, then centrifuged for 3 min at 14 000 g to separate the two phases. Aliquots of 100 μL were removed from *n*-butanol layer of each sample and placed in HPLC vials for analysis without evaporation. Serum MDA was then determined by injecting 20 μL of *n*-butanol extract onto an HPLC reverse phase column using a mixture of methanol and 50 mmol L^−1^ phosphate buffer, pH 6.7 (40̸60, *v̸v*) as mobile phase and detecting the MDA peak at 553 nm, excitation 515 nm. The concentration and identity of the eluted MDA was confirmed by comparison with a commercial standard and quantified by peak area using Agilent Technologies 1200 Series software (Santa Clara, CA, USA). All analysis was conducted in duplicate and data were displayed as the mean ± SEM [38].

## Result

Details of the demographic features and MDA for the ESRD patients and control group are shown in Table 2. ESRD patients had significantly higher MDA 2.07 (1.77–2.29) concentrations compared with control subjects 1.07 (0.89–1.26), (*p* < .001). Odd ratio and distribution of chemerin rs17173608 genotypes and alleles in ESRD patients and healthy controls after adjusted sex and age are shown in Table 3. Although no significant difference was monitored in genotypes frequencies of rs17173608 chemerin gene variant between ESRD patients and control group (χ^2^ = 3.9, df = 2, *p* = .14), but after adjusting the age and the sex, the OR value demonstrated that co dominant (T/G vs. T/T) genetic model significantly had a protective role against ESRD risk by a value of 0.504 (0.25–1.1, *p* = .037). Also, analyzing the dominant genetic model revealed that the presence of combined genotype of T/G + G/G had a significantly trend to decrease risk of ESRD with OR = 0.74 (0.5–1.1, *p* = .046). Distribution of the rs17173608 chemerin alleles in ESRD patients were significantly different from that in control group (χ^2^ = 3.6, df = 1, *p* = .047). As is shown in Table 3, the G allele of rs17173608 chemerin had a significantly trend to decrease the risk of ESRD to 0.79 (0.54–1.1, *p* = .048).

**Table 2.** Characteristics and distribution of risk factors in patients with end stage renal disease (ESRD) and control subjects in a population from Western Iran.

Parameter	Patients with ESRD (*n* = 136)	Control subjects (*n* = 137)	*p* Value
Age	58.1 ± 13.3	55.7 ± 7.3	.27
Gender (male/female)	89 (66.9%)/44 (33.1%)	85 (62%)/52 (38%)	.4
MDA (µM)	*2.07 (1.77–2.29)	*1.07 (0.89–1.26)	*<.001

* Median and interquartile range (IQR) for non-normally distributed data, and percentages for categorical data.

**Table 3.** Odd ratio and distribution of chemerin rs17173608 and alleles with respect to T/T or T and vaspin rs2236242 genotypes and alleles with respect to T/T or T respectively in ESRD patients after adjusted sex and age.

	ESRD patients (*n* = 110) OR (95% confidential interval)	Control subjects (*n* = 131)
Chemerin genotypes		
T/T	91 (82.7%)	95 (72.5%)
T/G	14 (12.7%)	29 (22.1%)
G/G	5 (4.5%) (χ^2^ = 3.9, df = 2, *p* = .14)	7 (5.3%)
Dominant		
T/G + G/G vs. T/T	(*n* = 19 (17.3%) vs. 91) 0.74 (0.5–1.1, *p* = .046)	(*n* = 36 (27.5%) vs. 95)
Codominant		
T/G vs. T/T	(*n* = 14 (13.3%) vs. 91) 0.504 (0.25–1.1, *p* = .037)	(*n* = 29 (23.4%) vs. 95)
Chemerin alleles		
T	*n* = 196	*n* = 219
G	0.79 (0.54–1.1, *p* = .048, *n* = 24) (χ^2^ = 3.6, df = 1, *p* = .047)	*n* = 43
Vaspin genotypes		
T/T	54 (49.1%)	66 (50.4%)
T/A	44 (40%)	51 (38.9%)
A/A	12 (10.9%) (χ^2^ = 0.05, df = 2, *p* = .98)	14 (10.7%)
Dominant model of vaspin		
T/T	54 (49.1%)	66 (50.4%)
T/A‏+A/A	56 (50.9%) (χ^2^ = 0.04, df = 1, *p* = .84) OR = 1.03 (0.8–1.3, *p* = .848)	65 (49.6%)
Vaspin alleles		
T	*n* = 152	*n* = 183
A	1.02 (0.84–1.3, *p* = .85, *n* = 68) (χ^2^ = 0.1, df = 1, *p* = .85)	*n* = 79

Odd ratio is an estimate relative risk for disease that was calculated and 95% confidence interval was obtained by using χ^2^ regression binary logistic analysis.

In addition, we compared distribution and odds ratios of the functional vaspin rs2236242 genotypes and alleles in ESRD patients and control group that are demonstrated in Table 3. We detected no significant association between vaspin rs2236242 genotypes and alleles in this study.

As Table 4 indicated, the MDA concentration in ESRD patients with T/G + G/G genotype was significantly higher compared with healthy subjects (2 (108–2.2) vs. 1.01 (0.9–1.2), *p* = .001) with the same genotype. In addition, similar results were observed concerning about vaspin A/A + T/A genotype in relation to MDA concentration in ESRD patients compared to control group (2.1 (1.8–2.3) vs. 1.1 (0.93–1.28), *p* = .001) (Table 5).

**Table 4.** Comparison of MDA concentration level between dominant model of chemerin genotypes (G/G/+ T/G vs. T/T) in ESRD subjects and control group.

Chemerin	ESRD patients	Control subjects	*p* Values
T/T MDA (µM)	2.04 (1.77–2.3)	1.06 (0.89–1.31)	<.001
T/G + G/G MDA (µM)	2 (108–2.2)	1.01 (0.9–1.2)	.001

A non-parametric Mann–Whitney *U*-test (median and interquartile range (IQR) for non-normally distributed data) was used to calculate the correlation value of serum MDA with dominant model of chemerin genotypes (G/G/+ T/G vs. T/T) patients and control subjects.

**Table 5.** Comparison of MDA concentration level between dominant model of vaspin genotypes (A/A + T/A vs. T/T) in ESRD subjects and control group.

Vaspin	ESRD patients	Control subjects	*p* Values
T/T MDA (µM)	2.1 (1.7–2.3)	1.05 (0.85–1.25)	<.001
A/A + T/A MDA (µM)	2.1 (1.8–2.3)	1.1 (0.93–1.28)	.001

A non-parametric Mann–Whitney *U*-test (Median and interquartile range (IQR) for non-normally distributed data) was used to calculate the correlation value of serum MDA with dominant model of chemerin genotypes (A/A + T/A vs. T/T) patients and control subjects.

To investigate the haplotypes of chemerin rs17173608 G allele and vaspin rs2236242 A allele in ESRD patients compared with control group, the web site of http://bioinfo.iconcologia.net/snpstats/start.htm was used and results were demonstrated in Table 6. We observed no significant association between chemerin rs17173608 G allele and vaspin rs2236242 A allele in ESRD patients and control group.

**Table 6.** Haplotype analysis between chemerin rs17173608 G allele and vaspin rs2236242 a allele in ESRD patients compared with control group.   Haplotype frequencies association with response (*n* = 241, crude analysis).

Chemerin	Vaspin	Group.Ca *n* (%)	Group.Co *n* (%)	OR (95% CI)	*p* Value
T	T	44 (40%)	49 (37.4%)	–	–
A	T	10 (9.1%)	17 (37.4%)	0.65 (0.27–1.6)	.65
T	G	47 (42.7%)	46 (35.1%)	1.07 (0.8–1.42)	.66
A	G	9 (8.2%)	18 (14.5%)	0.81 (0.6–1.1)	.16

Global haplotype association *p*-value: .41.

## Discussion

The present study for the first time has investigated the association between human chemerin rs17173608 and vaspin rs2236242 gene variants with the risk of ESRD and their correlation with plasma MDA level in Kurdish population from Western Iran. We have presented novel findings of the association between these SNPs with the risk of ESRD. Results of our study indicated that although the chemerin rs17173608 was not associated with ESRD, in dominant and codominant models, we detected that the presence of T/G + G/G vs. T/T and T/G vs. T/T genotypes had a significant trend to decrease the risk of ESRD. In fact, we suggest that the G allele of chemerin rs17173608 T/G may play a protective role against ESRD and decreased the ESRD susceptibility. Khaled et al. demonstrated that not only high level of serum chemerin could be a marker of diabetic nephropathy, but also chemerin gene rs17173608 polymorphism is associated with susceptibility to diabetic nephropathy in Egyptian patients. We could not find any study published in PubMed, Google scholar and other data bases in which the effect of vaspin rs2236242 T/A and chemerin rs17173608 T/G polymorphisms on the ESRD trigger or development had been investigated.

The high serum chemerin concentration in ESRD patients decreased to the values observed in healthy subjects after kidney transplantation [39]. Chemerin is expressed at high levels on immune cells and macrophages. Moreover, it acts as a chemotactic agent through its binding to chemerin receptor [11]. In humans, plasma chemerin concentrations are correlated with body mass index (BMI), glucose and lipid metabolism [7,16]. There are some evidences to suggest that chemerin is also expressed in animal kidneys [8]. Recently, the association between chemerin levels and obesity has been examined in hemodialyzed patients [40]. Studies on hemodialysis patients demonstrated that markers of kidney function are independently associated with plasma chemerin concentrations [41]. In the study performed by Blaszak et al., 70% higher chemerin concentration was observed in chronic kidney disease (CKD) patients compared to the control group (*p* < .001) and also elevated serum chemerin concentration in CKD patients was correlated to kidney function and hemodialysis treatment [16]. In addition, previous studies demonstrated that the high level of serum chemerin in CKD patients decreased to normal range after kidney transplantation [16,42]. A review study showed that the racial and ethnic differences have a straight and direct correlation with renal failure [43].

Current study demonstrated that, neither vaspin rs2236242 T/A genotypes nor alleles had a role in ESRD susceptibility in Kurdish population of Iran. The serum vaspin levels are reduced in Japanese chronic hemodialysis patients [44]. Although, serum vaspin levels were decreased in chronic hemodialysis (CD) patients in the study of Inoue, Seeger reported that the mean serum vaspin levels in chronic hemodialysis patients were similar to control groups [45]. Hida et al. have reported that high vaspin mRNA expression is associated with obesity and insulin-resistance in ESRD patients [46]. Moreover, two separate studies indicated a positive association between the elevated serum vaspin levels with obesity and impaired insulin sensitivity [47]. The higher vaspin levels in obesity and type 2 diabetes mellitus (T2DM) and its role in the progression of metabolic and glucose abnormalities have been emphasized in a meta-analysis [48]. The vaspin rs2236242 A/A genotype was associated with T2DM and with increased risk of disease in German KORA patients [20].

We demonstrated that ESRD patients who carry T/T and T/G + G/G genotypes of rs17173608 Chemerin gene had a significantly high serum MDA levels compared to control group with the same genotypes. A direct and straight association between chemerin with markers of oxidative stress and inflammation was demonstrated, whereas a negative association with the antioxidant status was found [49]. Yu et al. reported that chemerin may play an important role in initiation and development of obesity in T2DM patient and also in pathophysiology of insulin resistance, oxidative stress and inflammation [50].

## Conclusions

In the current study, for the first time, the association of chemerin rs17173608 G allele with decreased ESRD susceptibility has been indicated. Moreover, carriers of wild type and T/G + G/G genotypes of chemerin rs17173608 T/G had significantly high serum levels of MDA compared with healthy subjects. MDA serum concentration in ESRD patients was strongly higher than that in control group. In our studied population, the frequencies of genotypes and alleles of vaspin rs2236242 T/A in ESRD patients were similar to control group. Prospective studies with a larger sample size and different ethnicities are required to confirm our findings.

## Abbreviation

ESRD: End-stage renal disease
SNP: single nucleotide polymorphism
MDA: malondialdehyde
T-ARMS-PCR: tetra primer-amplification refractory mutation system-polymerase chain reaction

## References

[1] Aghighi M, Heidary Rouchi A, Zamyadi M, et al Dialysis in Iran. Iran J Kidney Dis. 2008;2:11–15. 19367003

[2] Bellomo R, Ronco C, Kellum JA, et al Acute renal failure–definition, outcome measures, animal models, fluid therapy and information technology needs: the Second International Consensus Conference of the Acute Dialysis Quality Initiative (ADQI) Group. Critical Care. 2004;8:R204. 15312219 10.1186/cc2872 PMC522841

[3] Barsoum RS. Chronic kidney disease in the developing world. N Engl J Med. 2006;354:997. 16525136 10.1056/NEJMp058318

[4] Yun Z, Yu-Ping Y, Zong-Wu T, et al Association of endothelial nitric oxide synthase gene polymorphisms with end-stage renal disease: a systematic review and meta-analysis. Renal Failure. 2014;36:987–993. 24673298 10.3109/0886022X.2014.900601

[5] Xue C, Nie W, Tang D, et al Apolipoprotein E gene variants on the risk of end stage renal disease. PLoS One. 2013;8:e83367. 24349494 10.1371/journal.pone.0083367 PMC3862680

[6] Müssig K, Staiger H, Machicao F, et al RARRES2, encoding the novel adipokine chemerin, is a genetic determinant of disproportionate regional body fat distribution: a comparative magnetic resonance imaging study. Metabolism. 2009;58:519–524. 19303973 10.1016/j.metabol.2008.11.011

[7] Bozaoglu K, Curran JE, Stocker CJ, et al Chemerin, a novel adipokine in the regulation of angiogenesis. J Clin Endocrinol Metab. 2010;95:2476–2485. 20237162 10.1210/jc.2010-0042 PMC2869547

[8] Roh S-g, Song S-H, Choi K-C, et al Chemerin-a new adipokine that modulates adipogenesis via its own receptor. Biochem Biophys Res Commun. 2007;362:1013–1018. 17767914 10.1016/j.bbrc.2007.08.104

[9] Fatima SS, Rehman R, Baig M, et al New roles of the multidimensional adipokine: chemerin. Peptides. 2014;62:15–20. 25278490 10.1016/j.peptides.2014.09.019

[10] Barnea G, Strapps W, Herrada G, et al The genetic design of signaling cascades to record receptor activation. Proc Natl Acad Sci. 2008;105:64–69. 18165312 10.1073/pnas.0710487105 PMC2224232

[11] Wittamer V, Franssen J-D, Vulcano M, et al Specific recruitment of antigen-presenting cells by chemerin, a novel processed ligand from human inflammatory fluids. J Exp Med. 2003;198:977–985. 14530373 10.1084/jem.20030382 PMC2194212

[12] Zabel BA, Allen SJ, Kulig P, et al Chemerin activation by serine proteases of the coagulation, fibrinolytic, and inflammatory cascades. J Biol Chem. 2005;280:34661–34666. 16096270 10.1074/jbc.M504868200

[13] Parolini S, Santoro A, Marcenaro E, et al The role of chemerin in the colocalization of NK and dendritic cell subsets into inflamed tissues. Blood. 2007;109:3625–3632. 17202316 10.1182/blood-2006-08-038844

[14] Ernst MC, Sinal CJ. Chemerin: at the crossroads of inflammation and obesity. Trends Endocrinol Metab. 2010;21:660–667. 20817486 10.1016/j.tem.2010.08.001

[15] Rourke J, Dranse H, Sinal C. Towards an integrative approach to understanding the role of chemerin in human health and disease. Obes Rev. 2013;14:245–262. 23216632 10.1111/obr.12009

[16] Blaszak J, Szolkiewicz M, Sucajtys-Szulc E, et al High serum chemerin level in CKD patients is related to kidney function, but not to its adipose tissue overproduction. Renal Failure. 2015;37:1033–1038. 25945605 10.3109/0886022X.2015.1040707

[17] Ernst MC, Issa M, Goralski KB, et al Chemerin exacerbates glucose intolerance in mouse models of obesity and diabetes. Endocrinology. 2010;151:1998–2007. 20228173 10.1210/en.2009-1098

[18] Jha V, Garcia-Garcia G, Iseki K, et al Chronic kidney disease: global dimension and perspectives. Lancet. 2013;382:260–272. 23727169 10.1016/S0140-6736(13)60687-X

[19] Leiherer A, Muendlein A, Kinz E, et al High plasma chemerin is associated with renal dysfunction and predictive for cardiovascular events – insights from phenotype and genotype characterization. Vascul Pharmacol. 2016;77:60–68. 26304698 10.1016/j.vph.2015.08.010

[20] Kempf K, Rose B, Illig T, et al Vaspin (SERPINA12) genotypes and risk of type 2 diabetes: results from the MONICA/KORA studies. Exp Clin Endocrinol Diabetes. 2010;118:184–189. 18726871 10.1055/s-2008-1081499

[21] Heiker JT. Vaspin (serpinA12) in obesity, insulin resistance, and inflammation. J Pept Sci. 2014;20:299–306. 24596079 10.1002/psc.2621

[22] Yan M, Su B, Peng W, et al Association of serum vaspin and adiponectin levels with renal function in patients with or without type 2 diabetes mellitus. J Diabetes Res. 2014;2014:868732. 25133192 10.1155/2014/868732 PMC4123592

[23] Nomani HH-NL, Aidy A, Vaisi-Raygani A, et al Association between GSTM1, GSTT1, and GSTP1 variants and the risk of end stage renal disease. Ren Fail. 2016;38:1455–1461. 27498857 10.1080/0886022X.2016.1214054

[24] Locatelli F, Canaud B, Eckardt KU, et al Oxidative stress in end‐stage renal disease: an emerging threat to patient outcome. Nephrol Dial Transplant. 2003;18:1272–1280. 12808161 10.1093/ndt/gfg074

[25] Nelson GJ, Morris VC, Schmidt PC, et al The urinary excretion of thiobarbituric acid reactive substances and malondialdehyde by normal adult males after consuming a diet containing salmon. Lipids. 1993;28:757–761. 8377591 10.1007/BF02536000

[26] Bahrehmand FV-RA, Kiani A, Rahimi Z, et al Matrix metalloproteinase-2 functional promoter polymorphism G1575A is associated with elevated circulatory MMP-2 levels and increased risk of cardiovascular disease in systemic lupus erythematosus patients. Lupus. 2012;21:616–624. 22323339 10.1177/0961203312436857

[27] Boaz M, Matas Z, Biro A, et al Serum malondialdehyde and prevalent cardiovascular disease in hemodialysis. Kidney Int. 1999;56:1078–1083. 10469377 10.1046/j.1523-1755.1999.00613.x

[28] Tanhapour M, Vaisi-Raygani A, Bahrehmand F, et al Association between the cytotoxic T-lymphocyte antigen-4 mutations and the susceptibility to systemic lupus erythematosus; Contribution markers of inflammation and oxidative stress. Cell Mol Biol (Noisy-Le-Grand) 2016;62:56–61. 10.14715/cmb/2016.62.12.10 27894401

[29] Tanhapour M, Miri A, Vaisi-Raygani A, et al Synergism between apolipoprotein E ε4 allele and paraoxonase (PON1) 55-M allele is associated with risk of systemic lupus erythematosus. Clin Rheumatol. 2018;37:971–977. 29273831 10.1007/s10067-017-3859-3

[30] Najafi K, Ahmadi S, Rahpeyma M, et al Study of serum malondialdehyde level in opioid and methamphetamine dependent patients. Acta Med Iran. 2017;55:616–620. 29228526

[31] Bahrehmand F, Vaisi-Raygani A, Kiani A, et al Matrix metalloproteinase 9 polymorphisms and systemic lupus erythematosus: correlation with systemic inflammatory markers and oxidative stress. Lupus. 2015;24:597–605. 25416694 10.1177/0961203314559085

[32] Asefi M, Vaisi‐Raygani A, Khodarahmi R, et al Methylentetrahydrofolatereductase (rs1801133) polymorphism and psoriasis: contribution to oxidative stress, lipid peroxidation and correlation with vascular adhesion protein 1, preliminary report. J Eur Acad Dermatol Venereol. 2014;28:1192–1198. 24118377 10.1111/jdv.12262

[33] Agarwal R, Chase SD Rapid, fluorimetric-liquid chromatographic determination of malondialdehyde in biological samples. J Chromatogr B Analyt Technol Biomed Life Sci. 2002;775:121–126. 10.1016/s1570-0232(02)00273-8 12101069

[34] Mohammadi Y, Vaisi-Raygani A, Shakiba E, et al Angiotensin II type 1 receptor A1166 C (rs5186) gene polymorphism increased risk and severity of psoriasis, contribution to oxidative stress, antioxidant statues, lipid peroxidation and correlation with vascular adhesion protein 1, preliminary report. J Eur Acad Dermatol Venereol. 2016;30:1395–1397. 26395033 10.1111/jdv.13241

[35] Hashemi M, Moazeni-roodi A, Bahari A, et al A tetra-primer amplification refractory mutation system–polymerase chain reaction for the detection of rs8099917 IL28B genotype. Nucleosides, Nucleotides Nucleic Acids. 2012;31:55–60. 22257210 10.1080/15257770.2011.643846

[36] Etlik O, Koksal V, Arican-Baris ST, et al Development and validation of a cost-effective in-house method, tetra-primer ARMS PCR assay, in genotyping of seven clinically important point mutations. Mol Cell Probes. 2011;25:177–181. 21530640 10.1016/j.mcp.2011.04.005

[37] Hashemi M, Rezaei H, Eskandari-Nasab E, et al Association between chemerin rs17173608 and vaspin rs2236242 gene polymorphisms and the metabolic syndrome, a preliminary report. Gene. 2012;510:113–117. 22982016 10.1016/j.gene.2012.08.048

[38] Asefi M, Vaisi‐Raygani A, Bahrehmand F, et al Paraoxonase 1 (PON1) 55 polymorphism, lipid profiles and psoriasis. Br J Dermatol. 2012;167:1279–1286. 22835076 10.1111/j.1365-2133.2012.11170.x

[39] Levey AS, Stevens LA, Schmid CH, et al A new equation to estimate glomerular filtration rate. Ann Intern Med. 2009;150:604–612. 19414839 10.7326/0003-4819-150-9-200905050-00006 PMC2763564

[40] Chen H-Y, Lin C-C, Chiu Y-L, et al Serum fetuin A and chemerin levels correlate with hepatic steatosis and regional adiposity in maintenance hemodialysis patients. PLoS One. 2012;7:e38415. 22815691 10.1371/journal.pone.0038415 PMC3398010

[41] Pfau D, Bachmann A, Lössner U, et al Serum levels of the adipokine chemerin in relation to renal function. Diabetes Care. 2010;33:171–173. 19837789 10.2337/dc09-1351 PMC2797967

[42] Rutkowski P, Sledzinski T, Zielinska H, et al Decrease of serum chemerin concentration in patients with end stage renal disease after successful kidney transplantation. Regul Pept. 2012;173:55–59. 21971116 10.1016/j.regpep.2011.09.005

[43] Freedman BI, Bowden DW. The role of genetic factors in the development of end-stage renal disease. Curr Opin Nephrol Hypertens. 1995;4:230–234. 7648217 10.1097/00041552-199505000-00005

[44] Imamura Y, Murayama N, Okudaira N, et al Prediction of fluoroquinolone-induced elevation in serum creatinine levels: a case of drug-endogenous substance interaction involving the inhibition of renal secretion. Clin Pharmacol Ther. 2011;89:81–88. 21124314 10.1038/clpt.2010.232

[45] Seeger J, Ziegelmeier M, Bachmann A, et al Serum levels of the adipokine vaspin in relation to metabolic and renal parameters. J Clin Endocrinol Metab. 2008;93:247–251. 17956947 10.1210/jc.2007-1853

[46] Hida K, Wada J, Eguchi J, et al Visceral adipose tissue-derived serine protease inhibitor: a unique insulin-sensitizing adipocytokine in obesity. Proc Natl Acad Sci USA. 2005;102:10610–10615. 16030142 10.1073/pnas.0504703102 PMC1180799

[47] Handisurya A, Riedl M, Vila G, et al Serum vaspin concentrations in relation to insulin sensitivity following RYGB-induced weight loss. Obes Surg. 2010;20:198–203. 19506980 10.1007/s11695-009-9882-y

[48] Feng R, Li Y, Wang C, et al Higher vaspin levels in subjects with obesity and type 2 diabetes mellitus: a meta-analysis. Diabetes Res Clin Pract. 2014;106:88–94. 25151227 10.1016/j.diabres.2014.07.026

[49] Fülöp P, Seres I, Lőrincz H, et al Association of chemerin with oxidative stress, inflammation and classical adipokines in non‐diabetic obese patients. J Cell Mol Med. 2014;18:1313–1320. 24702860 10.1111/jcmm.12282 PMC4124016

[50] Yu S, Zhang Y, Li M, et al Chemerin and apelin are positively correlated with inflammation in obese type 2 diabetic patients. Chin Med J. 2012;125:3440–3444. 23044303

